# Effects of nutritional low phosphorus status caused by extremely low phosphorus diet during lactation and early childhood on endochondral ossification in offspring rats

**DOI:** 10.3389/fnut.2026.1892725

**Published:** 2026-09-28

**Authors:** Jinyu Zhao, Yutong Xing, Huifeng Zhang, Yalei Pi, Ruichen Liu, Yibo Liu, Kemeng Chen, Yuqian Li, Yufei Liu, Yanan Zhang

**Affiliations:** Department of Pediatrics, The Second Hospital of Hebei Medical University, Shijiazhuang, China

**Keywords:** developmental origins of health and disease, growth plate, impaired bone mineralization, long bone development disorder, nutritional hypophosphatemic rickets, phosphorus deficiency

## Abstract

**Background:**

Fetal phosphorus is mainly deposited in bones in late pregnancy. Premature birth interrupts the supply of maternal phosphorus, combined with insufficient reserves, immature intestinal absorption and catch-up growth, the demand for mineralization surges, breast milk is difficult to meet, and it is easy to cause low-phosphorus bone disease; as the survival rate of premature infants increases, the incidence also increases.

**Objective:**

The core of the DOHaD theory is that the early living environment permanently reshapes the body through epigenetic mechanisms, thereby affecting long-term health and disease risks, based on the close relationship between the environment, health and disease at the molecular level. Based on the DOHaD theory, the rat model of perinatal continuous low phosphorus exposure confirmed that low phosphorus inhibited the endochondral ossification of long bones, which provided experimental basis for the prevention and treatment of low phosphorus rickets in premature infants.

**Methods:**

Pregnant SD rats were fed normal or very-low-phosphorus diet from gestation day 14–15 through lactation (3 weeks) and post-weaning (2 weeks). Offspring serum biochemistry, bone length, weight, and tibial histology (HE, toluidine blue, TRAP, Goldner, TUNEL), immunohistochemistry (RUNX2, COL1A1, COL2A1, VEGF, MMP13, OCN, BrdU, cleaved caspase-3), and Western blot (COL2A1, COL10A1, OCN) were assessed.

**Results:**

Low phosphorus caused serum biochemical changes (P↓, ALP↓; Ca↑, PTH↑, vitamin D↑), growth retardation, and altered protein expression (COL2A1, OCN↓, COL10A1↑; RUNX2, COL2A1, COL1A1, OCN, VEGF, BrdU, cleaved caspase-3↓, MMP13↑) in growth plate, indicative of defective chondrocyte functions. Histological examination showed that low phosphorus exposure led to the disorder of growth plate structure and inhibited endochondral ossification of long bone, which provided experimental basis for the prevention and treatment of low phosphorus rickets in premature infants.

**Conclusion:**

Maternal low-phosphorus diet induces hypophosphatemia and growth retardation in offspring, disrupts growth plate architecture, impairs chondrocyte proliferation and apoptosis, reduces bone mineralization and turnover.

## 1 Introduction

Phosphorus is the second most abundant mineral element in the human body, approximately 85% of which is stored in bones and teeth, mainly in the form of hydroxyapatite (1). It is essential for skeletal mineralization, maintenance of bone strength, cellular energy metabolism, and multiple physiological processes. During periods of rapid growth, phosphorus requirements increase substantially because of accelerated bone formation and mineral deposition (2). Preterm and very-low-birth-weight infants are particularly susceptible to phosphorus insufficiency because of reduced mineral stores at birth, rapid postnatal catch-up growth, and high nutritional requirements. In these infants, unfortified human milk alone may not provide sufficient phosphorus to support normal skeletal mineralization (3). Persistent phosphorus deficiency and associated hypophosphatemia are important contributors to metabolic bone disease of prematurity, although its pathogenesis is multifactorial and also involves calcium and vitamin D status, medication exposure, immobilization, and underlying disease (4). It not only impairs bone growth in children but may also induce a series of severe skeletal deformities (5). Prolonged phosphorus deficiency may impair hydroxyapatite formation and bone matrix mineralization, leading to excessive osteoid accumulation, deterioration of trabecular and cortical bone quality, and disturbed skeletal growth (6). In severe or persistent cases, defective mineralization may result in rachitic changes, skeletal deformities, and an increased risk of pathological fracture (7).

The epiphyseal growth plate is the principal site of endochondral ossification and plays a central role in longitudinal bone growth (8). Normal growth plate function depends on the coordinated proliferation, maturation, hypertrophy, and mineralization of chondrocytes. Disruption of these processes may impair cartilage-to-bone conversion and compromise skeletal development. However, direct investigation of the human growth plate is difficult. Growth plate biopsy is invasive and cannot be routinely performed in healthy children, whereas surgically obtained specimens are generally derived from pathological conditions and may not accurately represent normal physiological development (9). Ethical restrictions and limited tissue availability also make it difficult to obtain serial samples across different developmental stages (10). Although magnetic resonance imaging can provide non-invasive information on growth plate morphology, it cannot adequately resolve cellular-level alterations in chondrocyte organization and matrix mineralization (11). Histological examination provides valuable structural information but is limited to selected tissue sections and cannot support longitudinal observation in the same individual (12). In addition, growth plate morphology and cellular activity change dynamically with chronological age and skeletal maturation, making it difficult to distinguish physiological developmental changes from pathological alterations through cross-sectional clinical observations alone (13). Controlled animal models are therefore necessary for evaluating the effects of nutritional deficiency on growth plate development at defined developmental stages. Nevertheless, although the adverse effects of phosphorus deficiency on overall bone mineralization are well recognized, its influence on growth plate organization and endochondral ossification during continuous early-life exposure remains insufficiently understood.

The developmental origins of health and disease (DOHaD) theory was first proposed by Professor Barker in 1986 (14). The developmental origins of health and disease concept emphasizes that nutritional exposures during prenatal and early postnatal life may have lasting effects on organ development and subsequent health (15). Late gestation, lactation, and early postweaning life are critical periods of rapid skeletal growth and mineral accumulation. Maternal phosphorus restriction may reduce phosphorus availability to the fetus and suckling offspring, whereas continued restriction after weaning may prolong or aggravate its effects on the developing skeleton. Investigation of continuous phosphorus restriction across these developmental stages may therefore provide a more comprehensive understanding of the skeletal consequences of early-life phosphorus deficiency (16).

Accordingly, the present study established a rat model of continuous low-phosphorus exposure spanning late gestation, lactation, and early postweaning development. We investigated the effects of early-life phosphorus restriction on offspring growth plate morphology and bone mineralization, with particular emphasis on alterations in endochondral ossification. We hypothesized that sustained phosphorus restriction during critical developmental periods would disrupt growth plate organization and impair skeletal mineralization. This study may provide experimental evidence for understanding the developmental skeletal effects of early-life phosphorus deficiency and may contribute to the prevention and management of phosphorus deficiency-related bone disorders in infants and children (17).

## 2 Materials and methods

### 2.1 Animals and treatment

A total of 12 pregnant Sprague–Dawley rats (at 18–22 days of gestation, weighing 200–250 g, aged 8–9 weeks) were purchased from Liaoning Changsheng Biotechnology company limited by shares. The level of experimental animals: SPF (Specific Pathogen Free). All animal experimental procedures and protocols in this study were approved by the Ethics Committee of the Second Hospital of Hebei Medical University (Hebei, China) (Approval No.: 2025-AE478). All experimental animals used in this study were bred and raised at the Animal Experiment Center of the Second Hospital of Hebei Medical University. The groups were fed AIN-93G-based purified diets, purchased from Shulaibao (Wuhan) Biotechnology company limited. The rats were kept in a standard animal house at 22–25 °C under ventilation and hygienic conditions in a 12 h/12 h light/dark cycle and given free access to standard diet and water. Delivery after 1 week of adaptive feeding, the rats were divided into two groups: normal phosphorus content amino acid rat food feeding group and low phosphorus amino acid rat food feeding group. The other components (calcium content, total calorie value, etc.) except phosphate were the same between the two groups. All pups were nursed by their mother rats from postnatal day 0 to 21. After weaning on day 22, one offspring was randomly selected from each pregnant rat ‘s offspring to form two groups, with 6 male pups in each group, and continued to feed the corresponding rat food to 5 weeks of age. The pups were fed their respective diets until the end of the experiment on day 35. Six pregnant dams were assigned to each group, and three male pups and three female pups were randomly selected from each litter (*n* = 6 dams per group).

The specific composition of the two groups of feeds is shown in Table 1.

**Table 1:** Composition of the experimental diets.

Ingredient	Normal-phosphate diet (g/kg)	Very-low-phosphorus diet (g/kg)
L-Amino acid mixture	180.2	180.2
Corn starch	399.886	399.886
Sucrose	100.0	100.0
Maltodextrin	145.0	145.0
Soybean oil	70.0	70.0
t-Butylhydroquinone (tBHQ)	0.014	0.014
Cellulose	50.0	50.0
Mineral mix #210030	35.0	35.0
Sodium bicarbonate	7.4	7.4
Vitamin mix #310025	10.0	10.0
Choline bitartrate	2.5	2.5
Blue dye	0.05	0.05
Total phosphorus	0.6%	0.02%
Calcium	0.6%	0.6%
Total energy	4.0 kcal/g	4.0 kcal/g

### 2.2 Sample collection at sacrifice

Blood samples were collected from hearts for biochemical analysis. Following euthanasia, body weight was measured using an electronic balance. Nose-tail length and tibia length were then measured with a vernier caliper. Bilateral tibias of each animal were collected. The right tibia was used for histological evaluation (decalcification, paraffin embedding and sectioning), and the left tibia was frozen in liquid nitrogen and stored at-80 ° C for subsequent growth plate protein Western blot analysis. Right tibias were fixed in 4% paraformaldehyde for 4 °C overnight, EDTA (10%)-decalcified and paraffin-embedded (18). This distribution ensures that histological integrity is not affected by protein extraction. All histological measurements were consistent with the use of the right tibia to maintain consistency between the samples (Figure 1).

**Figure 1:**
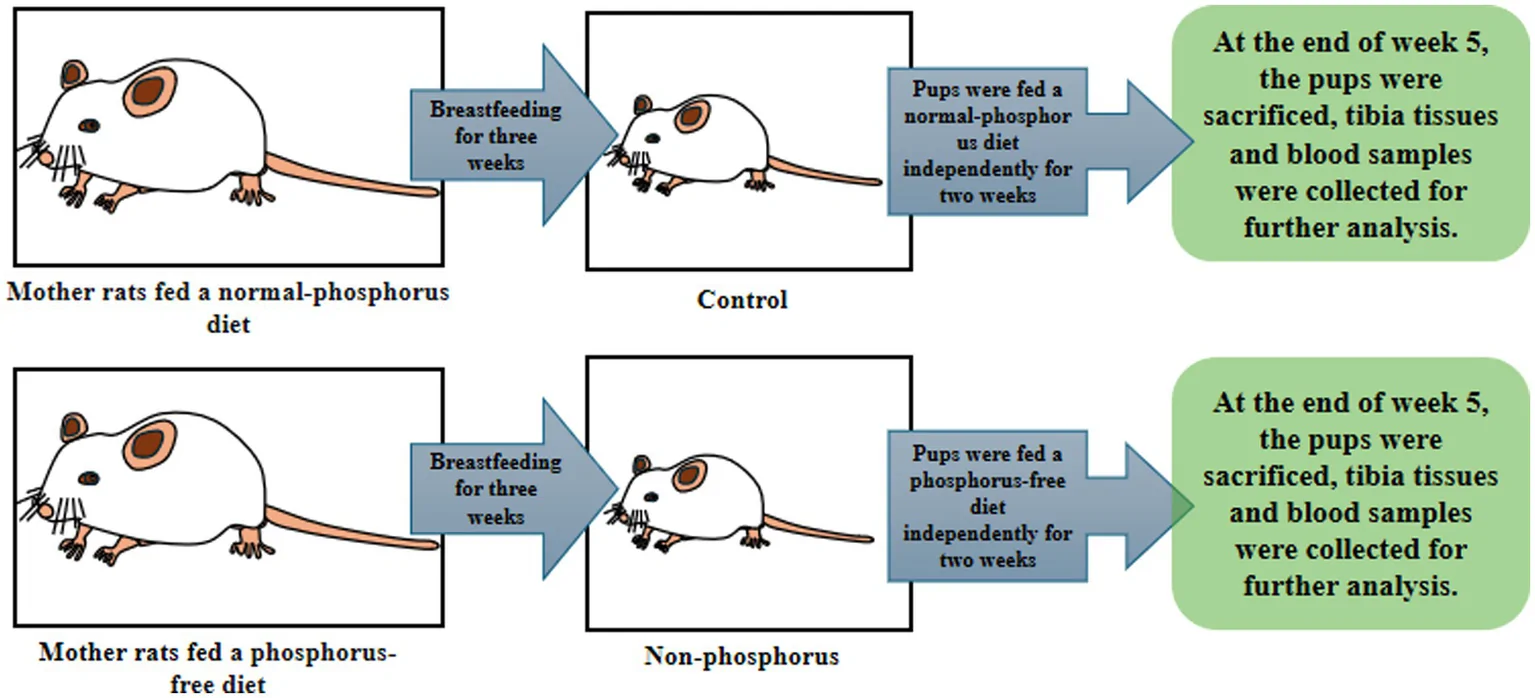
Schematic diagram of the experimental plan for modeling offspring rats.

### 2.3 Biochemical parameters

The levels of phosphorus (Solarbio, BC1655), calcium (Solarbio, BC0725), parathyroid hormone (Elabscience, E-ELR0535), active vitamin D (Elabscience, E-EL-0016) and alkaline phosphatase (Elabscience, E-EL-R0113) in serum were detected according to the instructions of the corresponding kits.

### 2.4 Tissue histology

The rat tibias were fixed in 4% paraformaldehyde for 4 °C overnight, EDTA (10%) – decalcified and paraffin-embedded. Then the samples were deparaffinized and rehydrated in xylene and graded ethanol, respectively. The 6-μm paraffin sections were stained with hematoxylin and eosin following standard protocols (19). For the quantitative evaluation of IHC and staining, we used 6 histological sections per group for both staining and IHC analyses. It is important to emphasize that each of these 6 sections was obtained from the right tibia of a different pup, and each pup was randomly selected from a different litter (one pup per litter). We randomly selected non-overlapping high-power fields (HPFs) per section. Then the software Image J was used to analyze the thickness of the growth plate and the width of each zone including proliferation zone and hypertrophy zone (20). The average proliferative zone (Pz) thickness and hypertrophic zone (Hz) thickness were calculated by measuring the center, medial quarter, and lateral quarter of the growth plate at the proximal tibia. The percentage proliferative zone thickness (%Pz) and percentage hypertrophic zone thickness (%Hz) were calculated relative to the thickness of the entire growth plate.

### 2.5 Immunohistochemistry

Immunohistochemistry was performed on paraffin-embedded tissue sections (6 μm). Sections were deparaffinized, rehydrated, and subjected to antigen retrieval using citrate buffer (pH 6.0) with a microwave heating method. Endogenous peroxidase activity was quenched by incubation in 3% H₂O₂. After blocking with 3% bovine serum albumin, sections were incubated overnight at 4 °C with primary antibodies, including COL2A1 antibody (1:100; GB11021-100, Servicebio), Cleaved-caspase3 antibody (1:500; GB11532-50, Servicebio), COL1A1 antibody (1:500; GB115707-50, Servicebio), MMP13 antibody (1:100; GB11247-50, Servicebio), VEGF antibody (1:200; GB15165-50, Servicebio), OCN (1:100; GB11233-50, Servicebio), RUNX2 antibody (1:1000; 20,700-1-AP, Proteintech) and BrdU antibody (1:100; GB12051, Servicebio), diluted in PBS. Subsequently, sections were incubated with HRP-conjugated secondary antibodies (1:500; GB23303, Servicebio) at room temperature for 50 min. Signal was developed using a 3,3′-diaminobenzidine (DAB) substrate kit, and nuclei were counterstained with hematoxylin. Finally, sections were dehydrated, cleared in xylene, and mounted with resin. Staining was visualized and imaged under a light microscope.

### 2.6 Western blot analysis

The relative protein expression levels were analyzed by western blotting. The tibia growth plate tissue samples were lysed with RIPA buffer containing protease inhibitor cocktail and phosphatase inhibitor. The mixture was then centrifuged at 12,000 g at 4 °C for 10 min. Then the protein concentrations were determined by a Bicinchoninic Acid Kit (Servicebio, G2026-200T). Meanwhile, the SDS loading buffer (5×) was then added into the supernatant for 10 min at 100 °C. A total of 10 μL protein within each lane were separated using 7.5%, 10 and 15% polyacrylamide gels and then transferred to a PVDF membrane. The membranes were first blocked by 5% defatted milk, and the various proteins were detected by incubation with primary antibodies, including COL2A1(1:1000; GB11021-100, Servicebio), COL10A1 (1:600; 26,984-1-AP, Proteintech), OCN (1:1000; ER61846, Huabio), GAPDH (1:10000; 10,494-1-AP, Proteintech) overnight at 4 °C. Next, the membranes were visualized by secondary antibody (1:10000; GB23303, Servicebio) conjugated by horseradish peroxidase (HRP) and ECL solution.

### 2.7 Goldner’s trichrome staining of paraffin sections

Goldner’s trichrome staining was performed on paraffin-embedded bone sections (6 μm) to distinguish mineralized bone, osteoid, and nuclei. Briefly, sections were deparaffinized and rehydrated (17). Nuclei were stained with a mixture of Goldner solutions A and B for 20 min, followed by differentiation in 1% acid alcohol. Subsequently, sections were sequentially stained in Solution C for 5–10 min, Solution D for 3 min under microscopic control, again in Solution C for 3–5 min, and finally in Solution E for 3–5 min. Each staining step was followed by a brief rinse in 0.2% acetic acid. Sections were then dehydrated, cleared in xylene, and mounted with neutral resin. Mineralized bone stained green, osteoid stained red, and nuclei appeared blue-purple.

For quantitative histomorphometric analysis, the following parameters were measured at the chondro-osseous junction and primary spongiosa region: bone volume per tissue volume (BV/TV), osteoid volume per bone volume (OV/BV), and osteoid volume per tissue volume (OV/TV).

### 2.8 Tartrate-resistant acid phosphatase staining

Tartrate-resistant acid phosphatase (TRAP) staining was performed on paraffin-embedded tissue sections (5 μm) to identify osteoclasts. Briefly, sections were deparaffinized, rehydrated through graded ethanol, and rinsed. The TRAP incubation working solution was freshly prepared by mixing reagents A, B, and C (Servicebio, G1050 kit) according to the manufacturer’s protocol. Sections were circled with a hydrophobic pen, placed in a humidified chamber, and covered with the filtered working solution. Incubation was carried out at 37 °C for 20 min. Subsequently, slides were rinsed and nuclei were counterstained with hematoxylin for 15 s, followed by differentiation and bluing. Finally, sections were dehydrated, cleared in xylene, and mounted with neutral resin. TRAP-positive osteoclasts were identified by dark red cytoplasmic staining and imaged under a light microscope.

### 2.9 Toluidine blue staining

Toluidine blue staining was performed to evaluate tibia growth plate structure. Deparaffinized and rehydrated tissue sections (6 μm thick) were stained with a 0.1% toluidine blue O solution (pH 4.0) for 8 min at room temperature. Slides were then briefly differentiated in 95% ethanol, dehydrated through absolute ethanol, and cleared in xylene. Finally, sections were mounted with a synthetic resin. Stained sections were examined under a light microscope, and images were captured using a digital camera system.

### 2.10 Evaluation of apoptosis

Apoptotic cells were identified by using a tunel kit (Service, G1504). After treatment proteinase K for 30 min at 37 °C, sections were incubated with the TUNEL reaction mixture for 2 h at 37 °C, rinsed, counterstained with DAPI staining solution (Service, G1012), and mounted with antifade mounting medium (Service, G1401).

### 2.11 Statistical analysis

The data are presented as mean ± standard deviation (SD). Error bars indicate the mean ± standard error of the mean (SEM). The n values in each graph represent biological replicates, that is, the number of individual animals in each group (*n* = 6). Each biological replicate sample was subjected to three independent technical replicates, and the results were averaged for statistical analysis. The unpaired students t-test was used to compare the data between two groups. A *p*-value <0.05 was considered statistically significant. The histogram was plotted using GraphPad Prism 10.0 software (19).

### 2.12 Sample size estimation

Sample size was estimated based on pre-experimental serum phosphorus data. The test efficiency was set to 80%, *α* = 0.05(double). The mean difference of sP between groups was 0.58 mmol/L, and the standard deviation was 0.4 mmol/L. It was calculated that at least 6 animals were needed in each group.

To assess the potential influence of sex, a two-way analysis of variance (ANOVA) was initially performed with diet and sex as independent variables for all parameters. The results showed that there was no significant interaction between diet and gender (all *p* > 0.05), indicating that the effect of diet intervention was consistent between genders, so the final analysis was combined. *p* < 0.05 was considered statistically significant. Therefore, 3 male pups and 3 female pups were set in each group.

## 3 Results

### 3.1 Construction of nutritional hypophosphatemic rickets model

A total of 12 pups completed the experiment, with 6 in the control group and 6 in the experimental group. At the fifth week, serum phosphorus concentration in low phosphorus group (control group: 2.53 ± 0.18 mmol/L, low phosphorus group: 0.60 ± 0.12 mmol/L; *p* < 0.0001) was significantly lower than that of the control group, indicating that the model of nutritional hypophosphatemic rickets was successfully constructed (see Figure 2A). A statistically significant difference in serum phosphorus levels was observed between the two groups (*P* < 0.0001).

**Figure 2:**
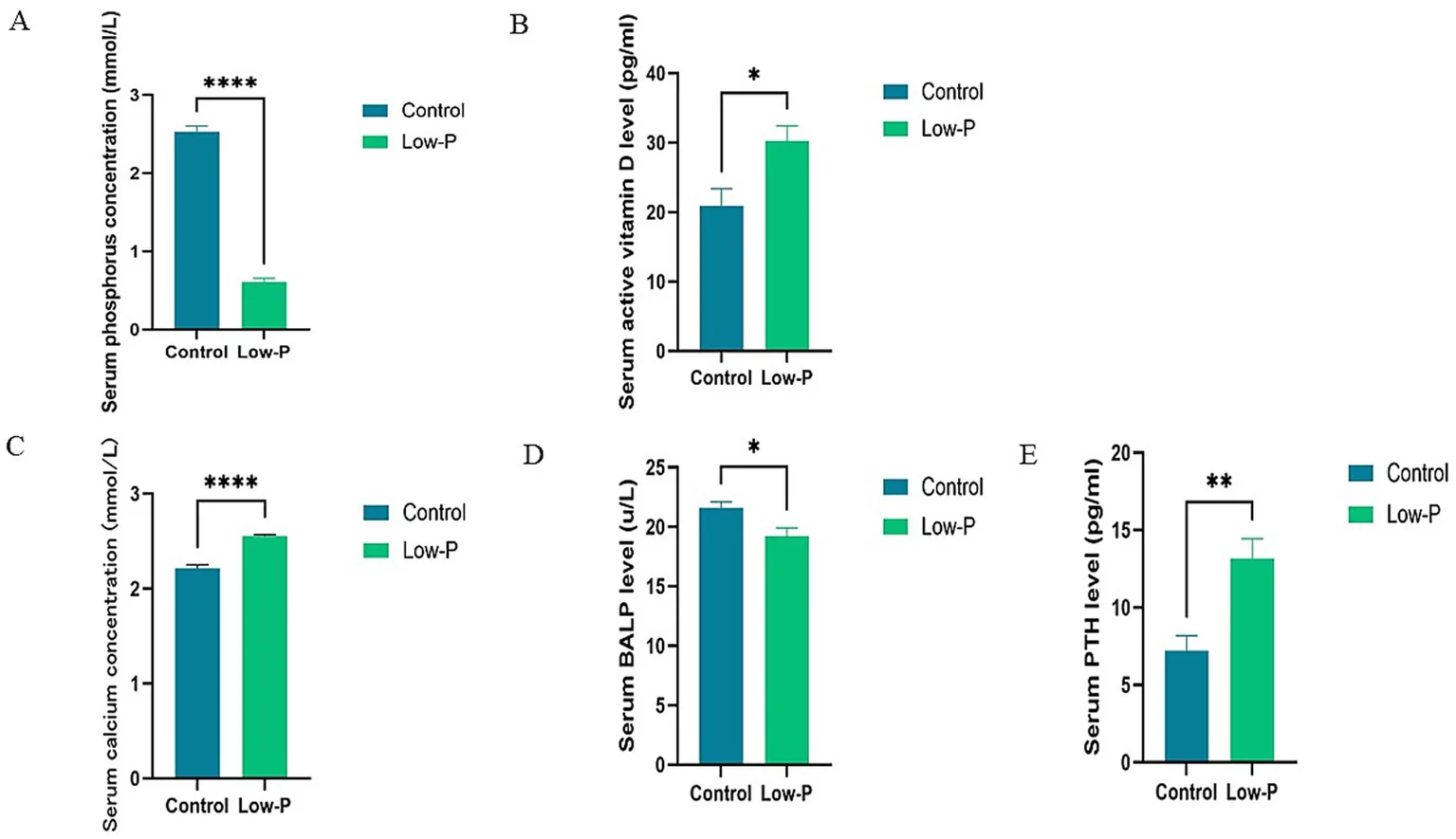
Effects of low phosphorus on serum phosphorus concentration, calcium concentration (*P* < 0.0001), active vitamin D and bone specific alkaline phosphatase levels (*P* < 0.05) in offspring rats. **(A)** Quantitative analysis of serum phosphorus concentration in offspring rats. **(B)** Quantitative analysis of serum active vitamin D levels in offspring rats. **(C)** Quantitative analysis of serum calcium concentration in offspring rats. **(D)** Quantitative analysis of serum alkaline phosphatase level in offspring rats. **(E)** Quantitative analysis of serum parathyroid hormone levels in offspring rats. Each group *n* = 6. Unpaired *t*-test was used for data analysis. *P < 0.1; **P < 0.01; ****P < 0.0001.

### 3.2 Biochemical analysis of calcium and phosphorus metabolism indexes and growth parameters

Serum alkaline phosphatase in low phosphorus group (control group 21.58 ± 1.24u/L, low phosphorus group 19.19 ± 1.76u/L; *p* < 0.05) (see Figure 2), body weight (control group 81.70 ± 4.81 g, low phosphorus group 19.83 ± 0.84 g; *p* < 0.0001), nasal tail length (control group: 26.27 ± 1.05 cm, low phosphorus group: 15.02 ± 0.29 cm; *p* < 0.0001), tibial length (control group: 2.20 ± 0.14 cm, low phosphorus group: 1.38 ± 0.15 cm; *p* < 0.0001) were significantly lower than the control group, serum calcium concentration (control group: 2.21 ± 0.09 mmol/L, low phosphorus group: 2.56 ± 0.03 mmol/L; *p* < 0.0001), serum active vitamin D level (control group: 20.87 ± 6.19 pg./mL, low phosphorus group: 30.24 ± 5.44 pg./mL; *p* < 0.05), serum parathyroid hormone levels (control group: 7.22 ± 2.35 pg./mL, low phosphorus group: 13.14 ± 3.17 pg./mL; *p* < 0.01) were higher than those in the control group (see Figure 3). The above differences were statistically significant.

**Figure 3:**
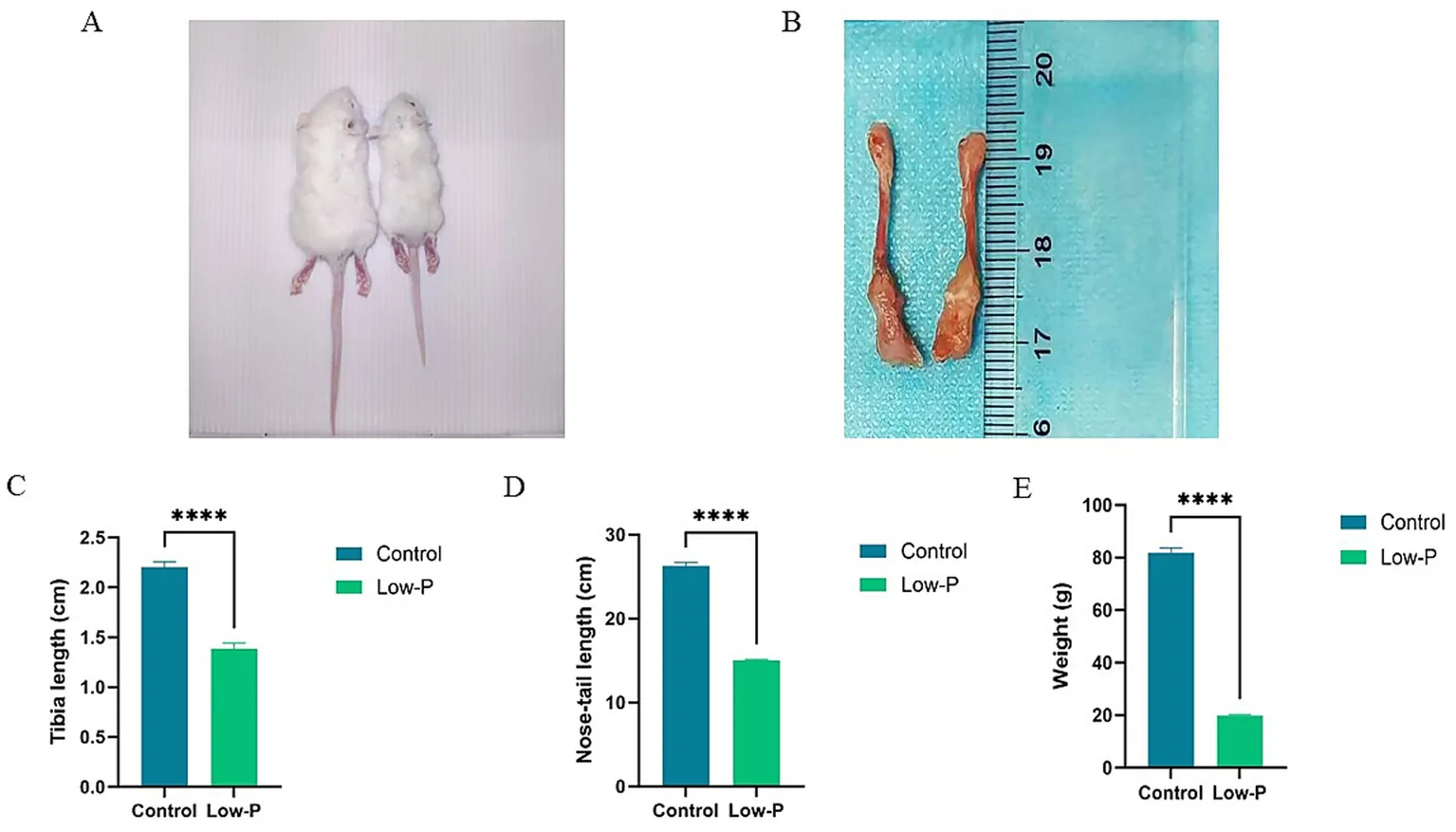
Effects of low phosphorus on body length, body weight, and tibia length in offspring rats (*P* < 0.0001). **(A)** General appearance of pups in control group and low phosphorus group at the end of the experiment. **(B)** Comparison of the appearance of the two groups of tibia. **(C)** Quantitative analysis of tibia length in pups. **(D)** Quantitative analysis of nose-tail length of pups. **(E)** Quantitative analysis of body weight of offspring; each group *n* = 6. Unpaired *t*-test was used for data analysis. ****P < 0.0001.

### 3.3 Growth plate morphology

The HE staining was performed on the tibial growth plate of 5-week-old offspring rats. The results showed that compared with the control group, the overall height of the growth plate in the low phosphorus group increased (control group: 1124.94 ± 41.93 μm, low phosphorus group: 1340.28 ± 71.84 μm; *p* < 0.0001) (Figure 4C). There was no significant difference in the proportion of the resting area of the growth plate to the growth plate (control group: 0.07 ± 0.03, low phosphorus group: 0.06 ± 0.04). The proliferation area of the growth plate was shortened (see Figure 4E), and the proportion of the growth plate was decreased (control group: 0.47 ± 0.02, low phosphorus group: 0.34 ± 0.02; *P* < 0.0001), hypertrophy area thickening (see Figure 4F), the proportion of growth plate increased (control group: 0.45 ± 0.02, low phosphorus group: 0.59 ± 0.07; *p* < 0.001), and the difference was statistically significant (21). The results of BrdU immunohistochemical staining showed that the positive signal of low phosphorus group was significantly lower than that of the control group (see Figures 5D–F), which also showed that the number of chondrocytes in the low phosphorus group was decreased (*P* < 0.001) (22). Moreover, we found from the staining results that the growth plate cartilage of the control group showed a uniform strip of thickness (see Figure 4A, D), while the growth plate morphology of the low phosphorus group was very abnormal, the boundary between the growth plate cartilage and the metaphyseal end bone was irregular (see Figure 4B), and some cartilage tissue even passed through the trabecular bone. In addition, under low phosphorus conditions, the distribution of chondrocytes in the growth plate was disordered, and the chondrocytes in the proliferative area no longer had a typical columnar structure, but gathered together, resulting in a decrease in the number and proliferation ability of chondrocytes, while the shape of hypertrophic chondrocytes changed from round to flat, the volume became smaller, the density increased, the polarity disappeared, and the irregular arrangement could not form an orderly growth column (Figure 4B). At the same time, the vascular infiltration of the low phosphorus group was destroyed, and the blood vessels could not be parallel to the growth column (Figure 4B). However, the chondrocytes in the control group still had obvious longitudinal polarity (Figure 4A). Toluidine blue staining showed that the hypertrophic area in the low phosphorus group was thickened, the staining band was abnormally widened and the boundary was unclear, extending into the metaphyseal end (see Figures 6A–D), the cartilage-bone junction was blurred or missing, and the cartilage tissue (purple red) protruded into the metaphyseal end area (forming a cartilage island).

**Figure 4:**
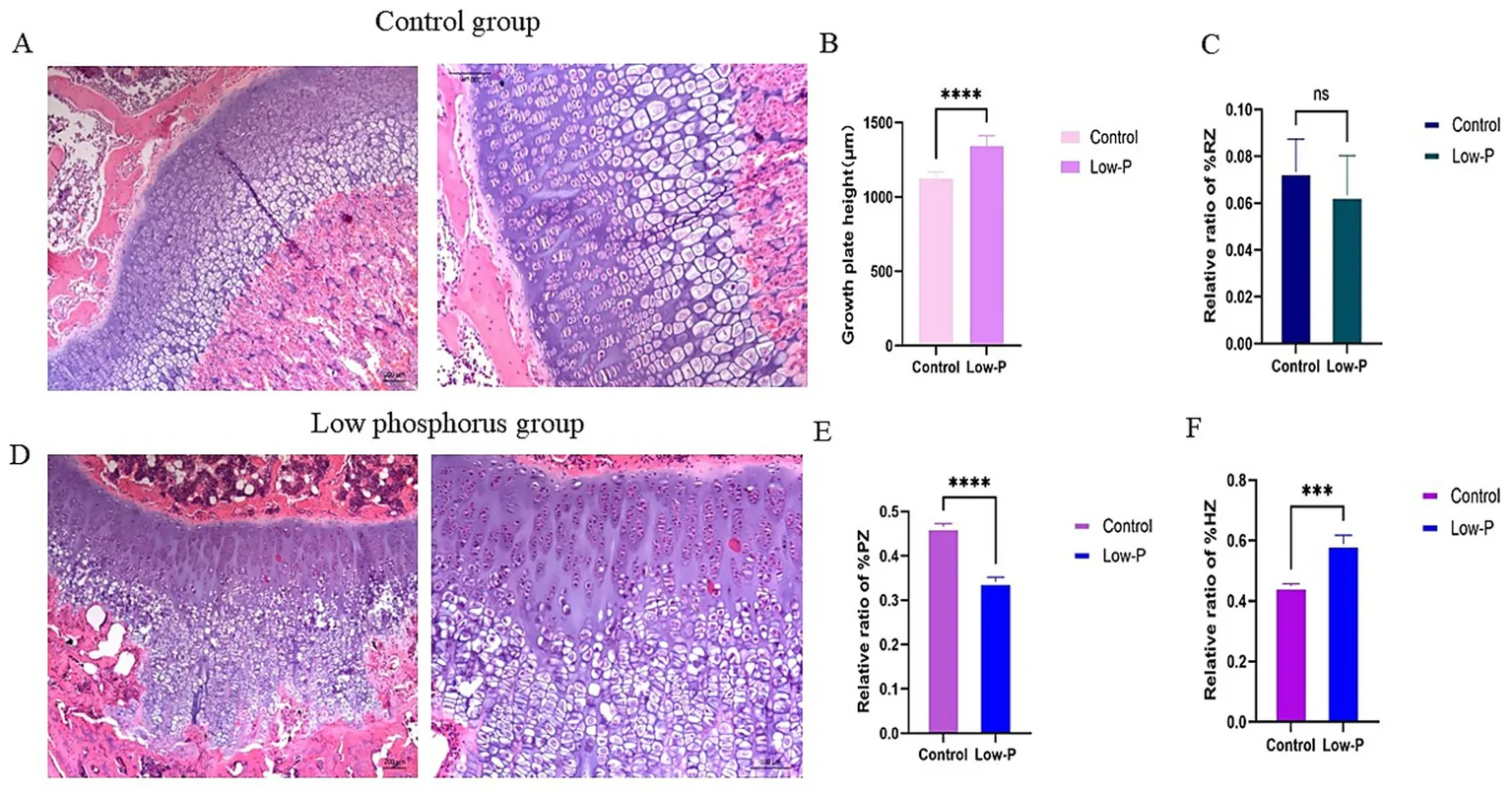
Morphological analysis of the tibia growth plate in offspring rats at postnatal week 5. **(A,D)** The representative images of HE stained sections of the proximal tibial growth plate of the control group and the low phosphorus group at 5 weeks under low magnification (40x) and high magnification (100x) were enlarged from left to right by 40 times and 100 times, respectively. **(B)** Growth plate height analysis of 5-week-old control group and low phosphorus group (RZ represents resting area; PZ represents the proliferation zone; HZ represents the hypertrophic zone). **(C)** The ratio of the resting area of the growth plate to the whole growth plate in the 5-week-old control group and the low phosphorus group. **(E)** The ratio of growth plate proliferation area to the whole growth plate in 5-week-old control group and low phosphorus group. **(F)** The ratio of the hypertrophic area of the growth plate to the whole growth plate in the 5-week-old control group and the low phosphorus group. ***P < 0.001; ****P < 0.0001.

**Figure 5:**
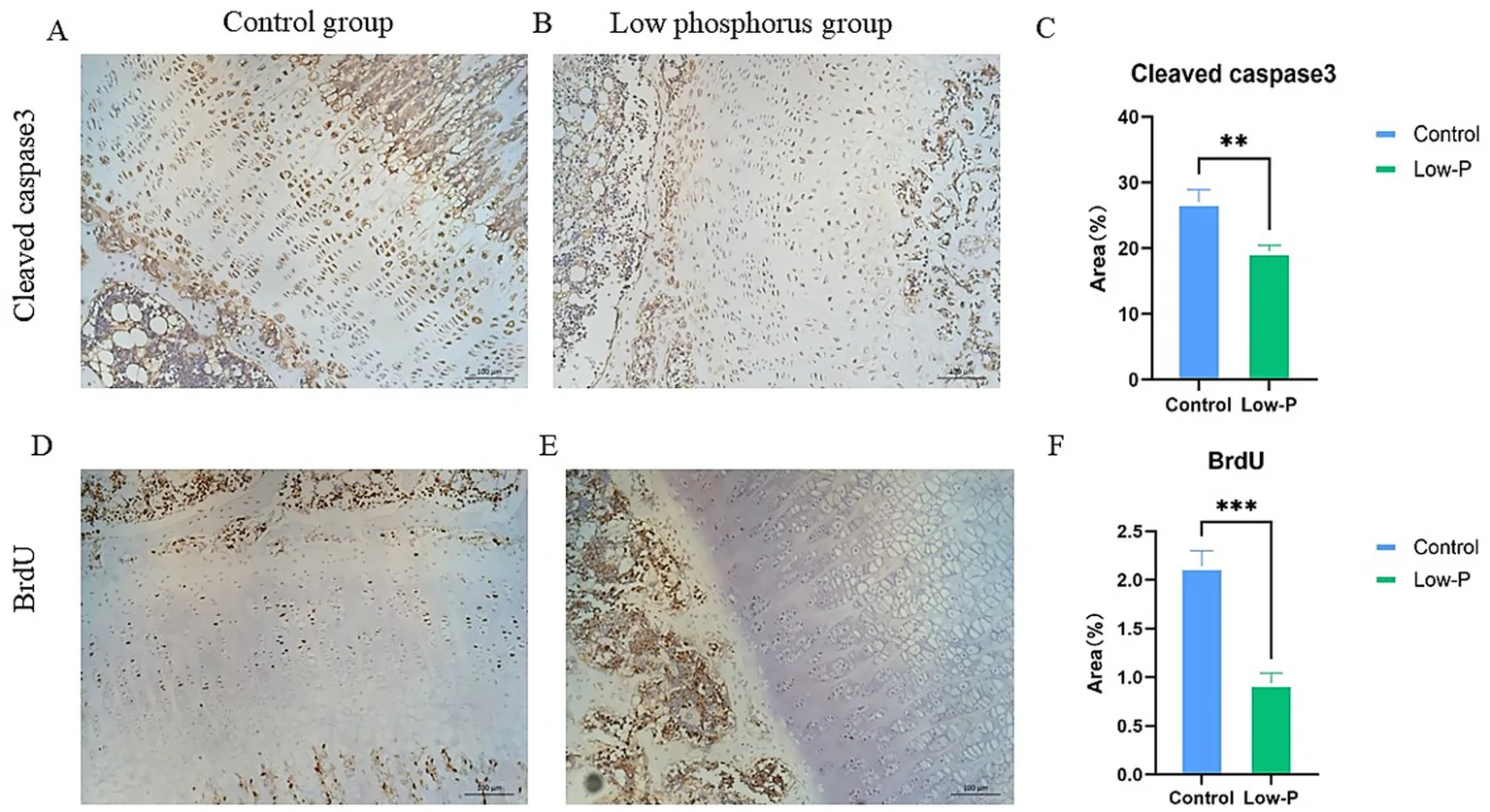
Immunohistochemical staining and semi-quantitative analysis of Cleaved Caspase-3 and BrdU in tibial growth plate at 5 W. The magnifications are100x. Panels **(A–C)** were the expression location and quantitative analysis of Cleaved Caspase-3 in the control group and the low phosphorus group. Panels **(D–F)** were the location and quantitative analysis of BrdU expression in the control group and the low phosphorus group (***P < 0.001; **P < 0.01; ns represents no statistical significance).

**Figure 6:**
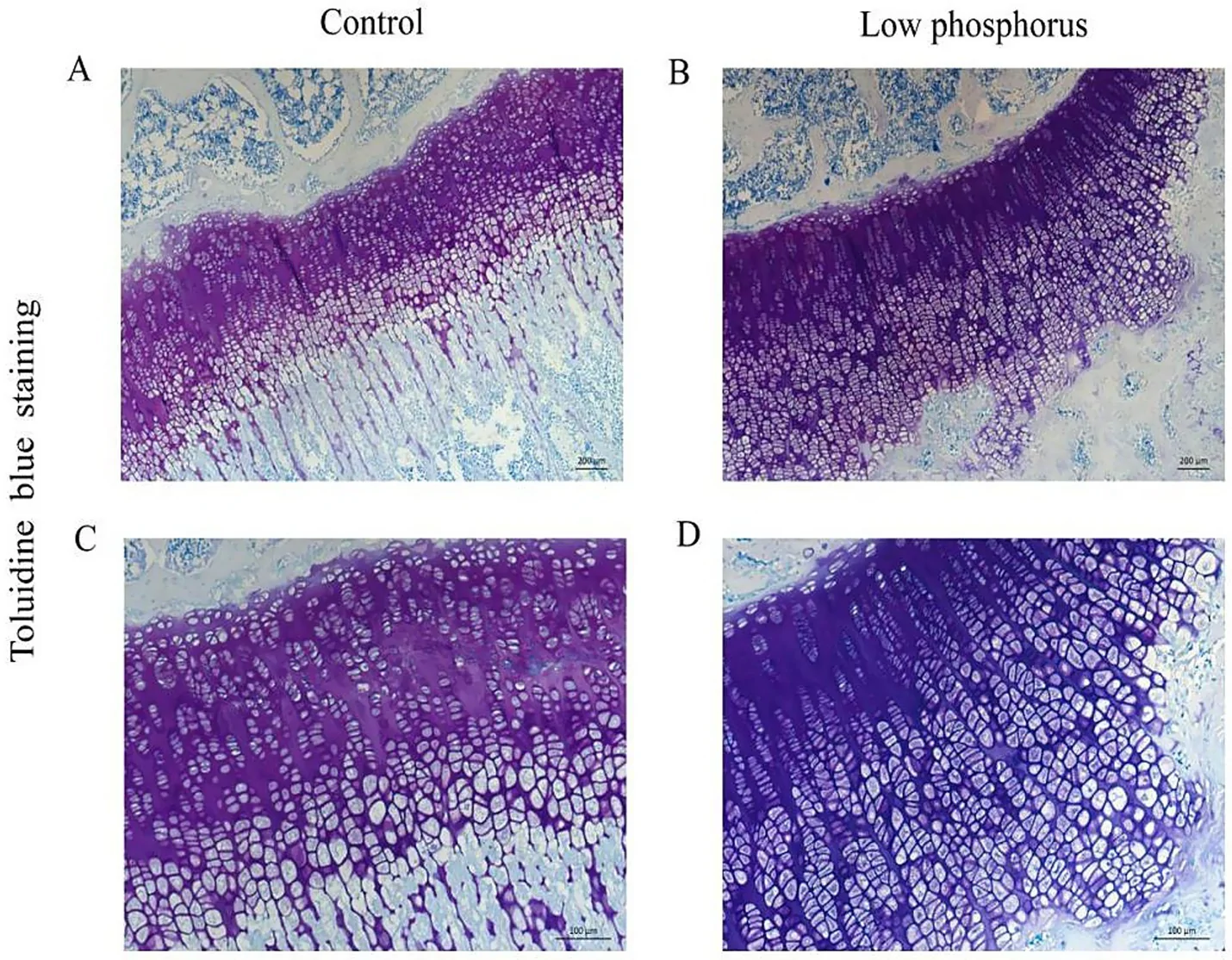
Representative images of toluidine blue staining of the tibial growth plate in offspring rats at postnatal week 5. The representative images of toluidine blue staining sections of the proximal tibial growth plate of the 5-week-old pups in the **(A,C)** control groups under low magnification (40x) and high magnification (100x), A is 40 times magnification, C is 100 times magnification; the representative images of toluidine blue staining sections of the proximal tibial growth plate of the 5-week-old pups in the **(B,D)** low phosphorus groups under low magnification (40x) and high magnification (100x), **(B)** is 40 times magnification, **(D)** is 100 times magnification.

### 3.4 Growth plate immunohistochemistry

It can be seen from Figure 5 that in the control cells, the expression of BrdU in the proliferation zone of the growth plate was typical, while in the proliferation zone of the low phosphorus group, the expression of BrdU was significantly reduced (see Figures 5D–F). The difference was statistically significant (*P* < 0.001) (23). In the control group, Cleaved Caspase-3 showed typical and strong expression in the hypertrophic zone of the growth plate. In contrast, the expression of Cleaved Caspase-3 in the hypertrophic zone of the growth plate in the low phosphorus group decreased significantly (see Figures 5A–C). The difference was statistically significant (*P* < 0.01) (24). Compared with the control group, the expression of OCN in the low phosphorus group was decreased (*P* < 0.001) (Table 2)(Figures 7D–F).

**Table 2:** Tibial growth plate immunohistochemistry.

Group	BrdU (%)	Cleaved caspase3 (%)	COL2A1 (%)	COL1A1 (%)	MMP13 (%)	RUNX2 (%)	VEGF (%)	OCN (%)
Control	2.14 ± 0.32	27.01 ± 4.70	53.93 ± 4.11	33.55 ± 4.22	22.38 ± 4.02	50.32 ± 5.53	21.48 ± 3.09	28.98 ± 3.85
Low phosphorus	0.94 ± 0.21	19.55 ± 2.18	18.29 ± 3.67	19.34 ± 2.01	32.28 ± 1.55	23.24 ± 2.78	12.39 ± 1.83	15.25 ± 1.85

The expression of COL1A1 in the low phosphorus group decreased (Figures 7A–C), indicating that the number or activity of osteoblasts decreased, and the synthesis and deposition of bone matrix decreased (*P* < 0.0001). The expression of VEGF in the hypertrophic area of the growth plate in the low phosphorus group decreased (*P* < 0.001). Osteogenic precursor cells and osteoclasts without blood vessels cannot initiate the subsequent osteogenic process (25), which is one of the core links of bone growth disorder caused by low phosphorus (see Figures 8A–C). In the low phosphorus group, the expression of RUNX2 in the trabecular bone under the proximal tibial growth plate was significantly lower than that in the control group (see Figure 8D–F, *P* < 0.0001).

**Figure 7:**
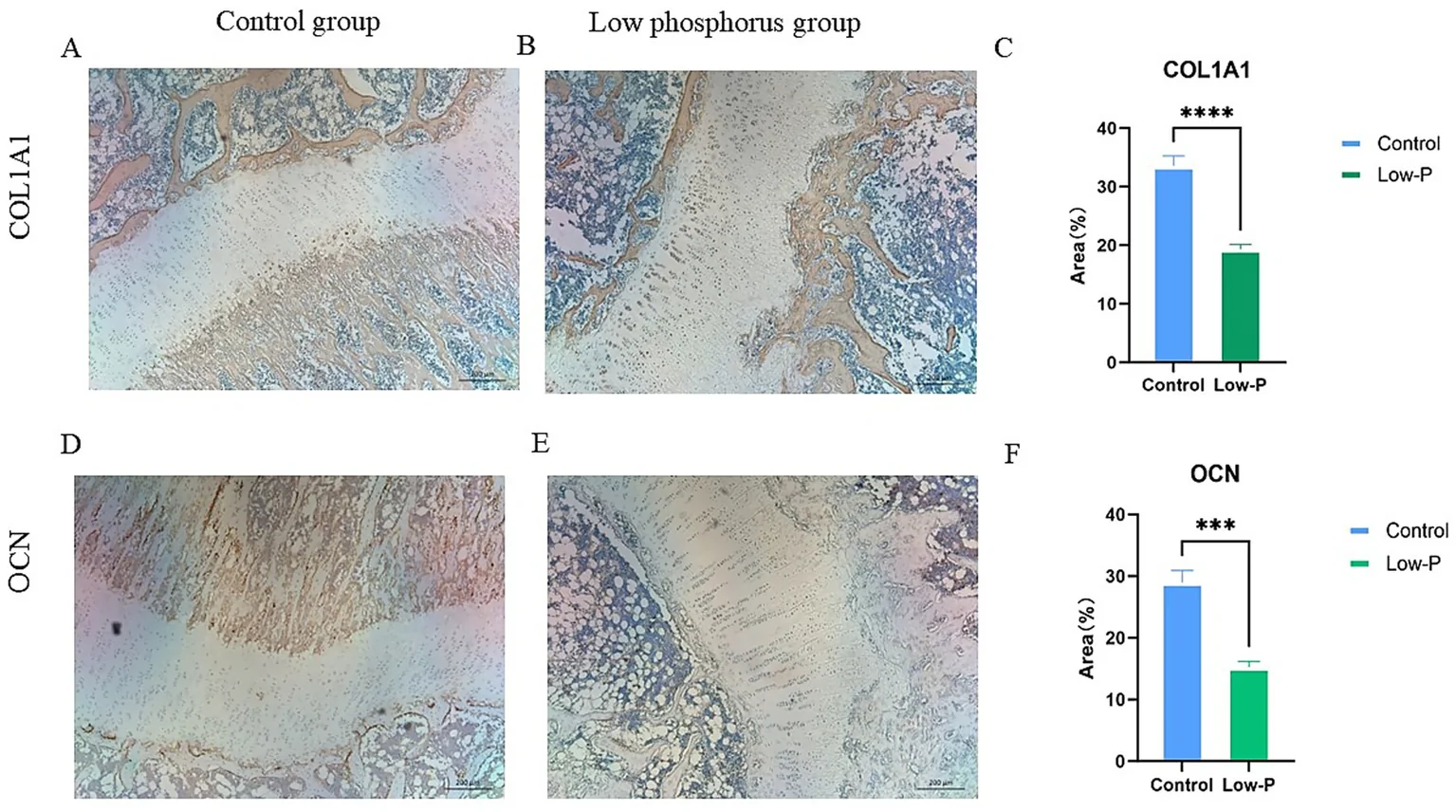
Expression of COL1A1, OCN in tibial trabecular bone. At the end of the experiment, the representative images of the immunohistochemical staining sections of the proximal tibial growth plate of the offspring were collected under low magnification (40×). Panels **(A–C)** were the control group and the low phosphorus group COL1A1 expression position and situation analysis; panels **(D–F)** were the control group and the low phosphorus group OCN expression position and situation analysis (****P < 0.0001; ***P < 0.001; *ns* represents no statistical significance).

**Figure 8:**
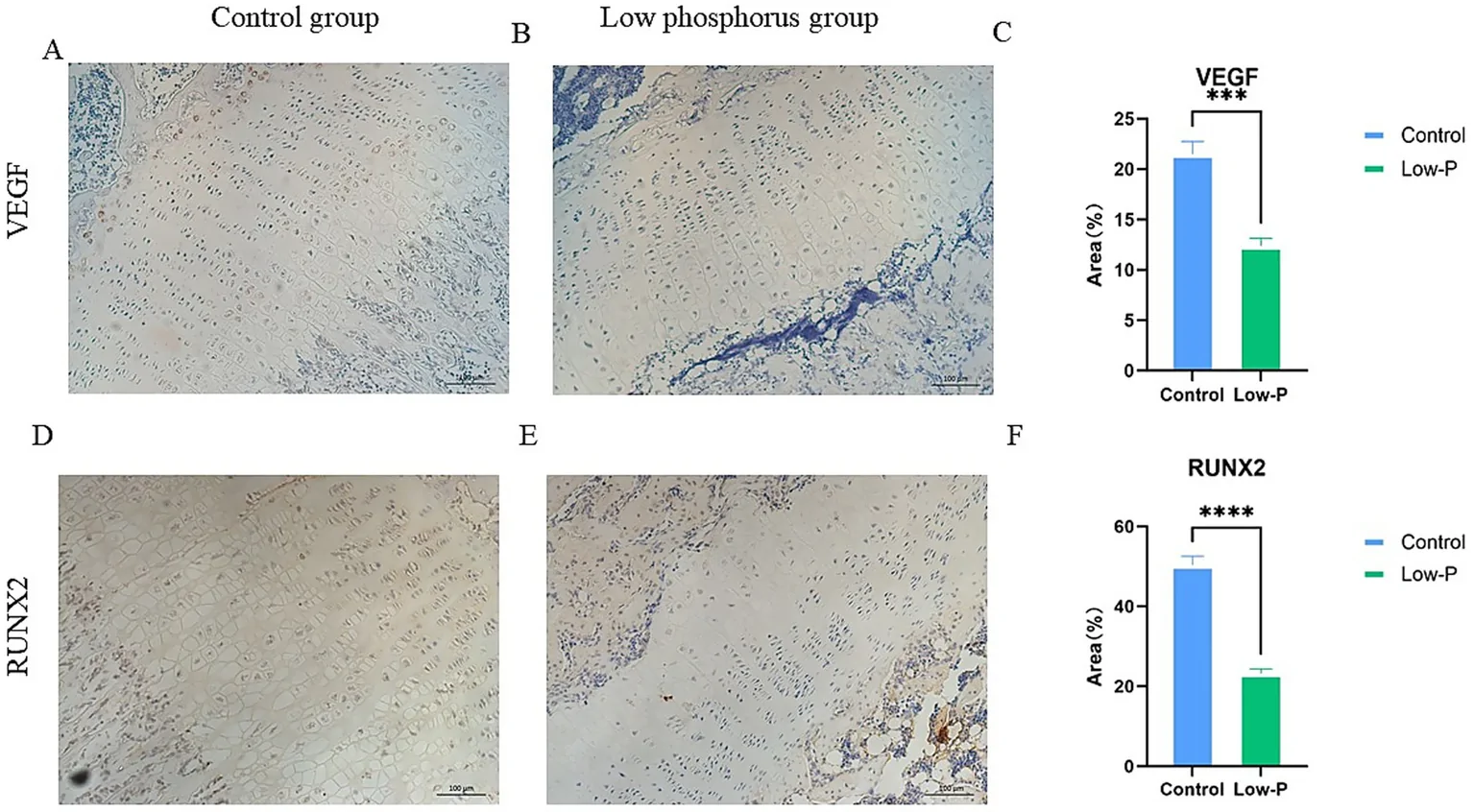
Immunohistochemical staining and semi-quantitative analysis of VEGF in the tibial growth plate and RUNX2 in the trabecular bone of the distal tibia at 5 weeks, at 100× magnification. At the end of the experiment, the representative images of the immunohistochemical staining sections of the proximal tibial growth plate of the offspring were collected under high magnification (100×). Panels **(A–C)** for the control group and low phosphorus group VEGF expression position and situation analysis; panels **(D–F)** were the expression position and situation analysis of RUNX2 in control group and low phosphorus group (****P < 0.0001; ***P < 0.001; *ns* represents no statistical significance).

By observing the distribution of COL2A1 expression, it was found that compared with the control group, the proliferation area of growth plate cartilage in the low phosphorus group decreased, the number of chondrocytes in the proliferative state decreased (see Figures 9A–C), and the expression of type 2 collagen decreased (*P* < 0.0001). Compared with the control group, the expression of MMP13 in the hypertrophic area of the tibial growth plate in the low phosphorus group was increased (26) (*P* < 0.001) (Figures 9D–F). The increased expression of MMP13, excessive decomposition of bone collagen matrix, increase bone mineralization defects and bone destruction (27). Hypertrophic chondrocytes are dysfunctional and cannot effectively degrade the surrounding cartilage matrix. In addition, we found that compared with the control group, the distribution pattern of the above genes in the low phosphorus group changed, which was characterized by reduced expression and patchy. The expression is easily detected in the upper hypertrophic region, but is scattered in the lower region, especially in the most irregular region.

**Figure 9:**
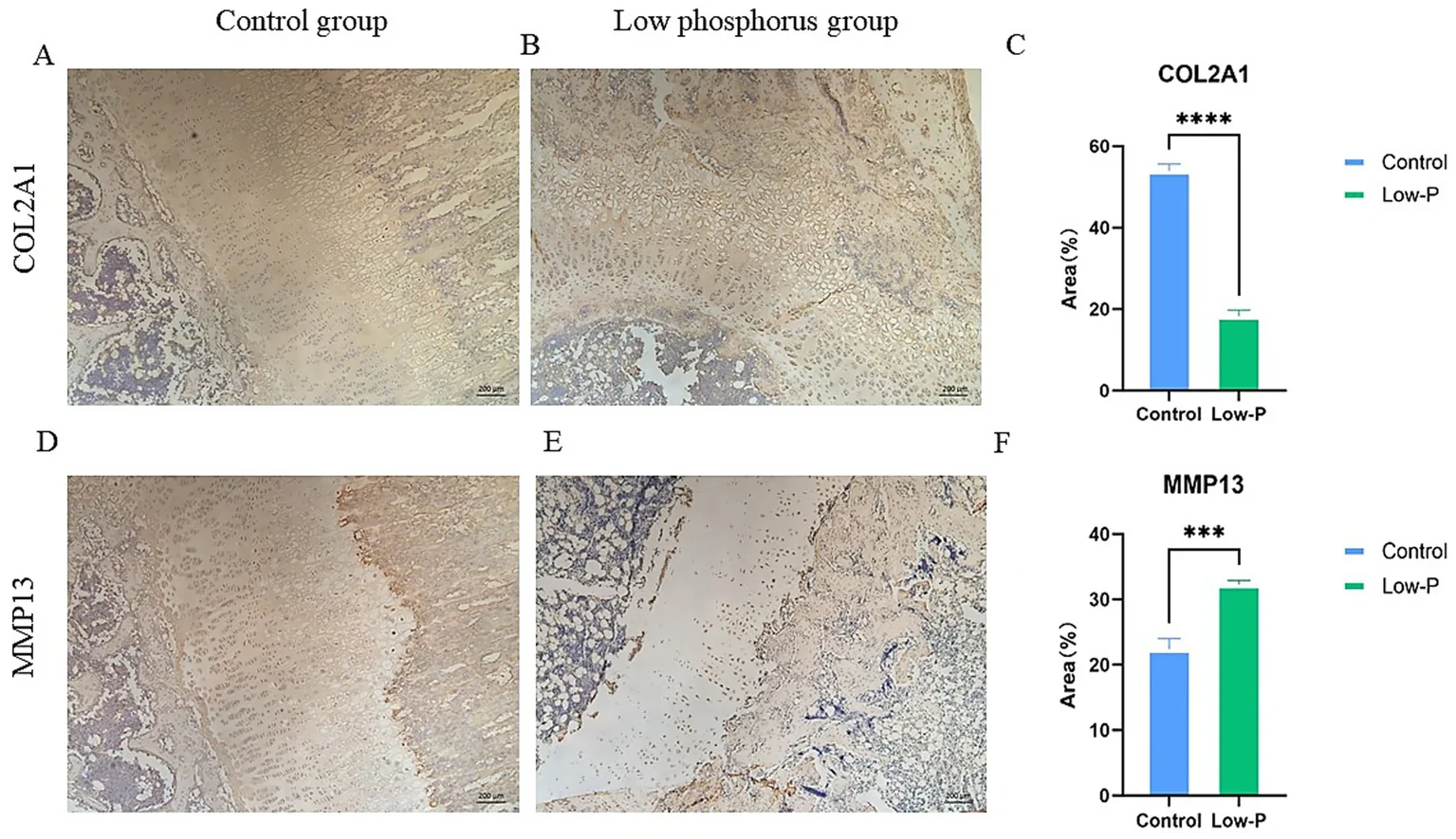
Immunohistochemical staining and semi-quantitative analysis of COL2A1 and MMP13 in tibial growth plate at 5 W. The magnifications are 100x. At the end of the experiment, the representative images of the immunohistochemical staining sections of the proximal tibial growth plate of the offspring were collected under low magnification (40×). Panels **(A–C)** were the control group and the low phosphorus group COL2A1 expression position and situation analysis; panels **(D–F)** were control group and low phosphorus group MMP13 expression position and situation analysis (****P < 0.0001; ***P < 0.001; *ns* represents no statistical significance).

### 3.5 Western blot

Western blot analysis of growth plate cartilage tissue (Figure 10) showed that the protein expression levels of COL2A1 and OCN in the low phosphorus group was lower than that in the control group (*P* < 0.05), and the protein expression level of COL10A1 was up-regulated (*P* < 0.05).

**Figure 10:**
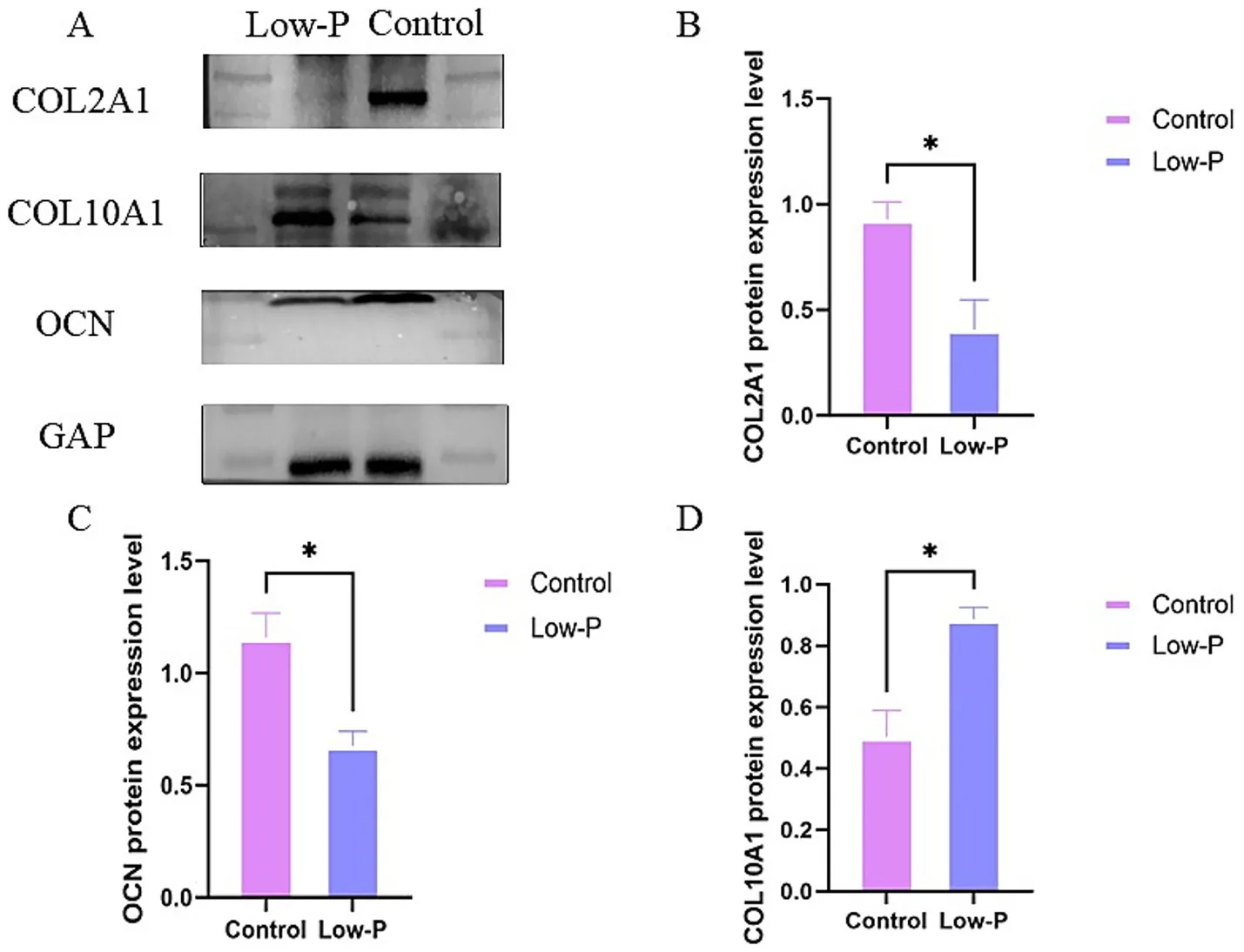
**(A)** Western blot bands of COL2A1, COL10A1, OCN and GAPDH; **(B-D)** quantitative analyses of COL2A1, OCN and COL10A1, respectively. *P < 0.1.

### 3.6 Bone mineralization

Compared with the control group, the activity of osteoclasts was significantly reduced in the low phosphorus group (28) (*P* < 0.01) (Figures 11A,C,E,G) (31). Furthermore, their distribution within the trabecular bone was altered, under low phosphorus conditions, these cells were no longer uniformly scattered along the cartilage-bone junction but instead showed a more irregular and dispersed pattern (29) and focal aggregation of these cells was observed in certain regions of the growth plate (Figures 11B,D,F). Consistent with these findings, Goldner’s trichrome staining (Figure 12) revealed a similar pattern. Quantitative histomorphometric analysis of Goldner-stained sections revealed that the low-phosphorus group had a significantly lower BV/TV compared with the control group (*P* < 0.05), indicating reduced cancellous bone mass. Conversely, both OV/BV and OV/TV were significantly increased in the low-phosphorus group (OV/BV: *P* < 0.01; OV/TV: *P* < 0.01), indicating excessive osteoid accumulation and impaired mineralization (Figures 12E–G).

**Figure 11:**
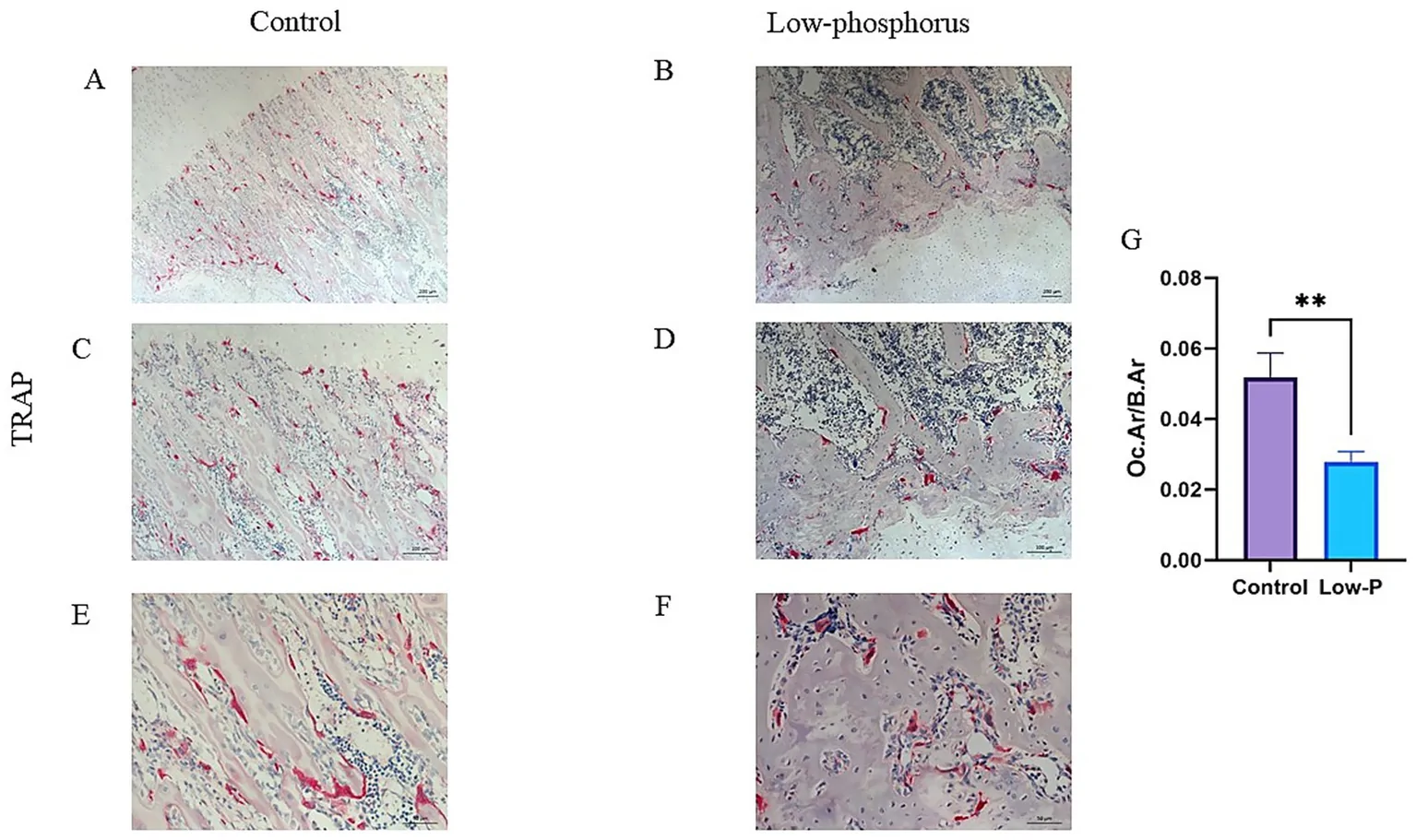
TRAP staining of tibial trabecular bone of rats in control group and experimental group at 5 W. From upper to lower are magnifications of 40x, 100x and 200x, respectively. Panels **(A,C,E)** represent the control group, while panels **(B,D,F)** represent the low phosphorus group. Panel **(G)** represents the percentage of TRAP positive area in the total area of bone tissue. **P < 0.01.

**Figure 12:**
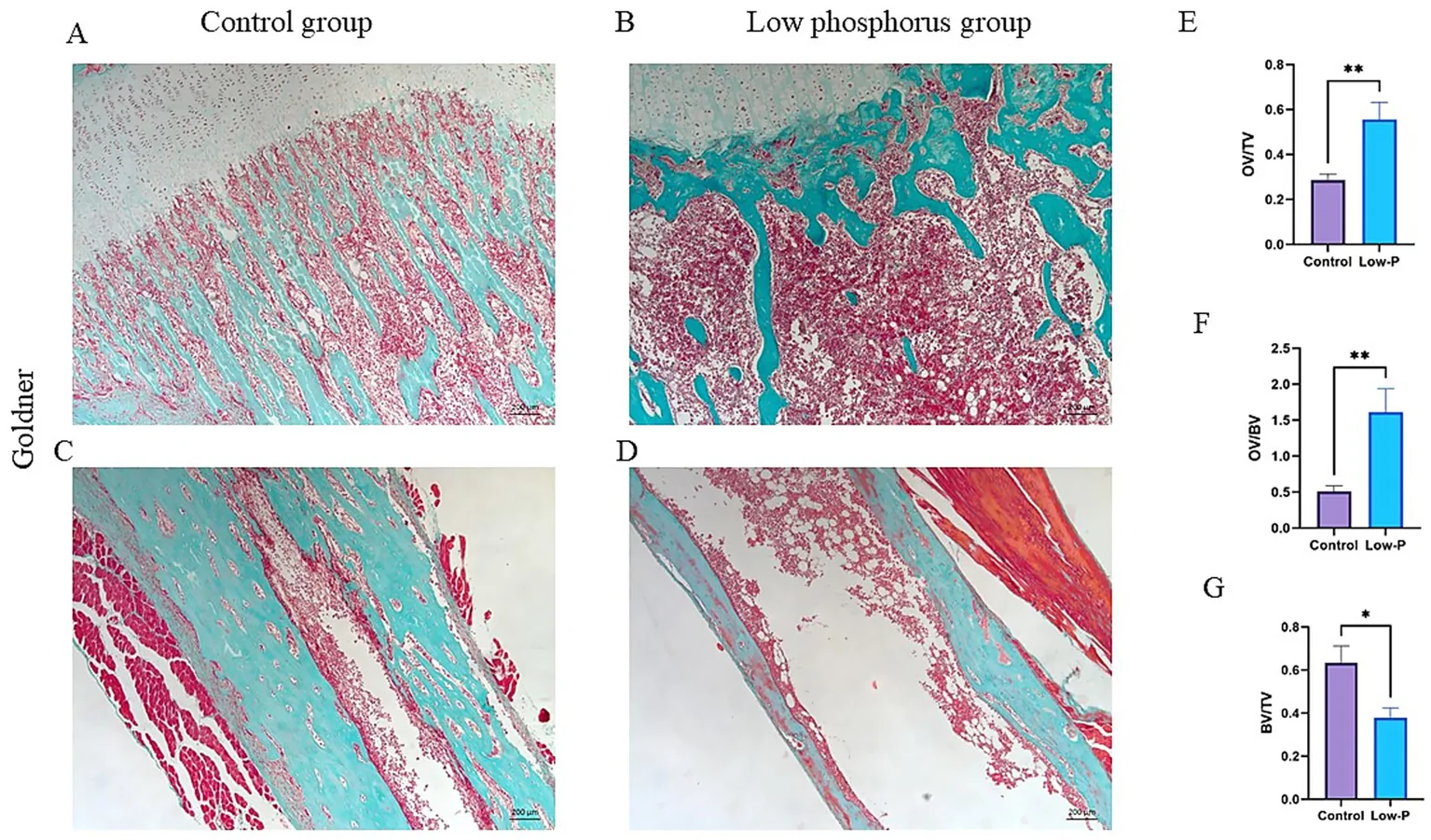
Representative images of Goldner’s staining of the trabecular bone and cortical bone in the tibia at 5 weeks of age. **(A,B)** Goldner trichrome staining of the trabecular bone area of the tibia in the control group and the experimental group at 5 weeks, with a magnification of 40 times. **(C,D)** The control group and the experimental group of tibial cortical bone backbone of the Goldner trichrome staining, magnification of 40 times. **(E)** OV/TV; **(F)** OV/BV; **(G)** BV/TV. *P < 0.1; **P < 0.01.

In the control group, the mineralized bone was evenly distributed at the bone-cartilage junction and showed a regular strip in the longitudinal direction. In contrast, the mineralized bone in the low phosphorus group was irregularly agglomerated and arranged in disorder. In addition, cortical bone mineralization was significantly reduced in the low phosphorus group.

### 3.7 Apoptosis

Compared with the control group (30), the low phosphorus group exhibited weakened apoptotic signals in both the growth plate and trabecular bone regions compared with the control group (Figure 13). This result was consistent with the immunohistochemical findings for cleaved caspase-3 (Figures 5A–C) (31).

**Figure 13:**
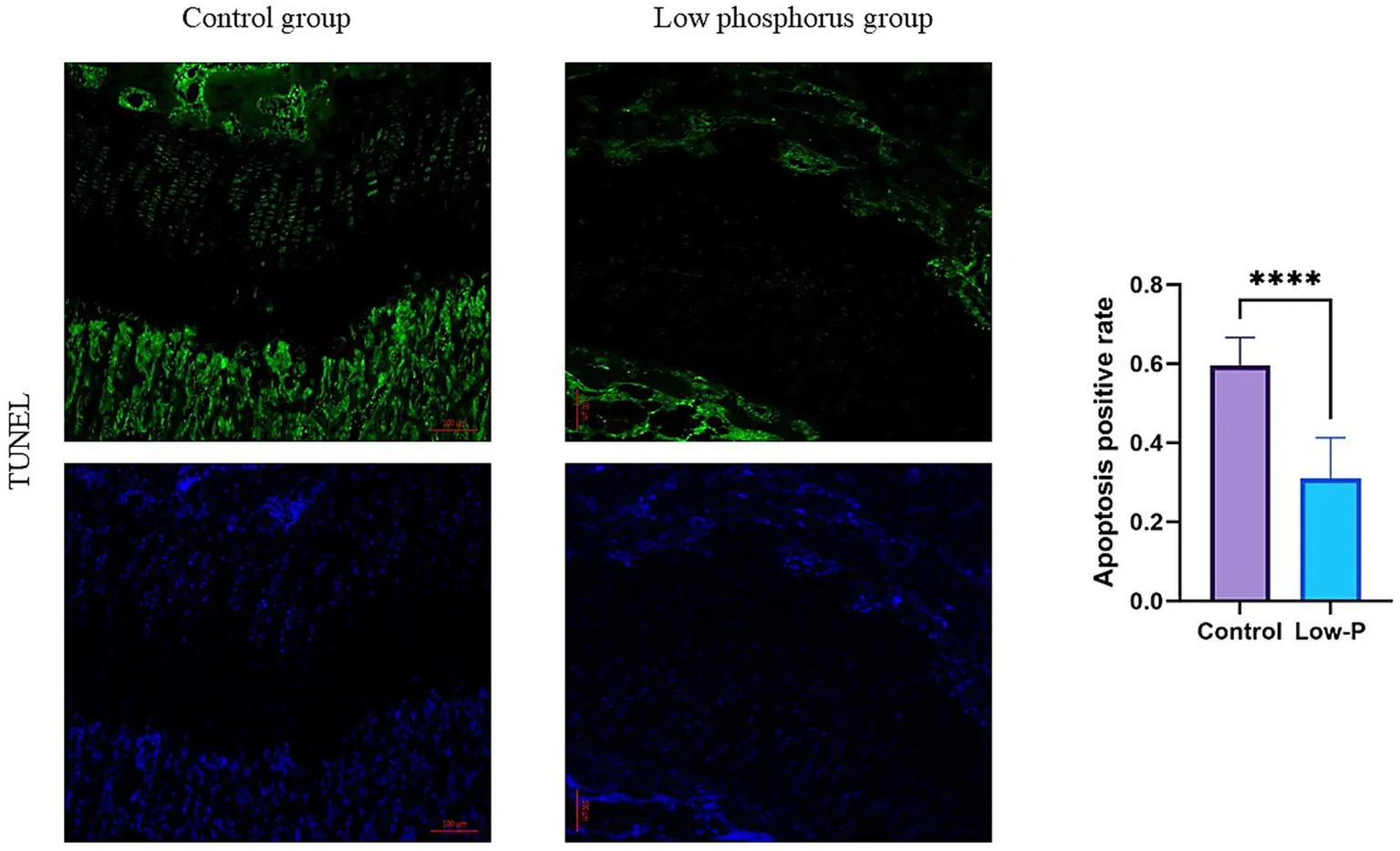
Representative images of TUNEL staining in the tibial growth plate at 5 weeks, at 100 × magnification. ****P < 0.0001.

## 4 Discussion

Nutritional hypophosphatemic rickets is a kind of metabolic disorder disease with abnormal phosphorus level in blood as the main manifestation. The pathogenesis is complex and seriously endangers the growth and development of children. Appropriate phosphorus level is a key factor affecting the growth and development of organisms. Previous studies have shown that chondromatosis is not a direct result of decreased VDR function, but is accompanied by hypocalcemia, hypophosphatemia and hyperparathyroidism (32). Therefore, phosphorus balance plays a very important role in bone development (33). Hypophosphatemia-induced terminal hypertrophic chondrocyte apoptosis disorder is an important medium for the occurrence of nutritional hypophosphatemic rickets. Phosphorus is an essential element for bone mineralization and growth and development. When it is insufficient, it will cause a series of pathological changes, such as rickets, osteomalacia, and growth retardation. It is worth noting that since the development origin of health and disease (DOHaD) theory was proposed by Barker (34), its evidence chain has long been highly concentrated in the programming mechanism of metabolic, cardiovascular and endocrine systems (such as insulin resistance, obesity, hypertension and dyslipidemia). In contrast, the skeletal system, as the largest “metabolically active organ” and “mechanical support structure” of the human body, has received insufficient attention under this theoretical framework, forming a knowledge gap that needs to be filled in the field. Traditional DOHaD bone-related studies mostly focused on the static endpoint measurement of bone mineral density or bone mineral content after birth, or only regarded bone changes as an “incidental phenomenon” of calcium and phosphorus metabolism disorders, rather than a target organ independently regulated by early life programming mechanisms (35). In this study, the dynamic process of development, i.e., the time series events of endochondral ossification of growth plate, was used to break through the two limitations of previous studies. First, the exposure paradigm of DOHaD was expanded from the classic “overnutrition and maternal high-fat diet” to “the restrictive supply of key minerals (phosphorus) during the development window period”. Secondly, the observation dimension of DOHaD was expanded from the traditional “energy metabolism and soft tissue homeostasis”(such as fat, liver, blood vessels) to the “bone tissue development and growth plate endochondral ossification microenvironment,” focusing on the dynamic remodeling process of bone structural integrity and bone-cartilage interface. This study shows that low phosphorus can interfere with chondrocyte proliferation, hypertrophy differentiation and clearance, vascular-matrix remodeling and terminal mineralization, suggesting that low phosphorus bone injury is centered on the obstruction of the whole sequence of endochondral ossification. For the first time, the programming effect of perinatal low phosphorus exposure was revealed from the three levels of molecule-cell-tissue, which provided experimental basis for the prevention and treatment of low phosphorus rickets in premature infants and the application of DOHaD theory in early bone intervention.

We successfully established a lactating mother-offspring rat model of continuous low phosphorus exposure during lactation and early childhood. However, it should be clearly pointed out that this model is an extreme state model, because there is no complete low phosphorus in our daily diet. In the future, we need to further verify the results of this experiment under different low phosphorus concentration gradients. This study was designed to cover three levels: late pregnancy, lactation and early independent diet stage of young rats. It can not only simulate a single cause (nutrient phosphorus deficiency), but also simulate long-term cumulative damage (36). This process captures multiple important growth processes such as embryonic tissue, organ differentiation, rapid growth to late “catch-up growth.” Therefore, phosphorus deficiency exists both in the “basic programming” process of bone development and in the “execution period” of bone development. On this basis, we explored the regulatory effect of phosphorus deficiency on chondrocyte osteogenic differentiation and bone tissue homeostasis in bone growth plate, and provided new ideas for the prevention and treatment of low phosphorus rickets in premature infants caused by insufficient phosphorus supplementation. Intrachondral bone formation begins with the accumulation of mesenchymal cells and then differentiates into chondrocytes. During the growth and development of the embryo and later period, the proliferated chondrocytes differentiate into hypertrophic chondrocytes, which undergo programmed cell death, namely apoptosis, and then are replaced by new bone tissue (37).

We observed a decrease in serum phosphorus and alkaline phosphatase levels in the low phosphorus group, while serum calcium, parathyroid hormone and active vitamin D levels increased, accompanied by a decrease in body weight, nasal tail length and tibia length. The above results suggest that persistent nutritional low phosphorus status not only causes the disorder of calcium and phosphorus metabolism in the whole body, but also further affects the longitudinal growth of long bone and the development of bone tissue, and finally shows an obvious growth retardation phenotype (38).

The longitudinal growth of long bone depends on the orderly proliferation, differentiation, hypertrophy and apoptosis of chondrocytes in the growth plate (39). In this study, HE and toluidine blue staining showed that the growth plate of the low phosphorus group was widened as a whole, the proliferation area was shortened, the hypertrophy area was widened, the columnar arrangement of chondrocytes was disordered, the polarity was lost, and the cartilage tissue was abnormally protruded into the metaphysis to form a “cartilage island” like change. It is suggested that the shortening of the limbs of offspring rats caused by phosphorus deficiency may be related to the shortening of the proliferative zone and the decrease of chondrocyte apoptosis in the hypertrophic zone. At the same time, BrdU positive signal decreased and COL2A1 expression decreased, the decreased COL2A1 protein level observed in the low-phosphorus group may reflect impaired chondrocyte proliferation and reduced extracellular matrix synthesis. This interpretation is supported by the concurrent reduction in BrdU-positive cells, suggesting that phosphorus availability is critical for maintaining the proliferative capacity of growth plate chondrocytes. However, the precise molecular mechanisms by which phosphate regulates COL2A1 expression warrant further investigation. “The results of Cleaved caspase-3 and TUNEL showed that the apoptosis of hypertrophic chondrocytes was weakened, and the expression of COL10A1 was increased, indicating that abnormal accumulation of hypertrophic chondrocytes occurred under low phosphorus condition. The above changes indicate that the growth retardation caused by low phosphorus is not simply manifested as insufficient mineralization, but is accompanied by the imbalance of growth plate chondrocyte dynamics, that is, insufficient proliferation and obstruction of clearance after hypertrophy, resulting in abnormal growth plate structure and delayed endochondral ossification.

Further analysis showed that low phosphorus also had a significant inhibitory effect on the endochondral bone turnover. The expression of VEGF and MMP13 suggested that the ability of hypertrophic chondrocytes to promote vascular invasion and cartilage matrix degradation and remodeling was also abnormal (40). The “attraction signal” from cartilage to bone was weakened. This leads to a decrease in angiogenesis and cannot effectively invade the hypertrophic cartilage area. Under normal circumstances, after the apoptosis of hypertrophic chondrocytes, blood vessels, osteogenic precursor cells and osteoclasts enter the cartilage-bone junction to complete the conversion of cartilage template to bone tissue; in the low phosphorus state, this process is blocked, resulting in abnormal retention of hypertrophic cartilage area, irregular boundary of cartilage-bone junction, and eventually forming histological manifestations of delayed osteogenesis in cartilage (41). In addition to changes in growth plates, low phosphorus further impaired osteogenesis and mineralization. The results of immunohistochemistry and Western blot showed that the expression of RUNX2, COL1A1 and OCN decreased, suggesting that osteoblast differentiation activity, bone matrix synthesis and mineralization maturation were inhibited (42). The expression of RUNX2 was down-regulated and MMP13 was up-regulated in hypophosphatemia, suggesting that the differentiation and functional activity of osteoblasts decreased, and the synthesis ability of bone matrix was also reduced. RUNX2 reduces the inhibition of osteogenic differentiation and matrix synthesis such as type I collagen and osteocalcin, resulting in blocked bone formation; the increase of MMP13 is driven by low phosphorus activation of NF-κB / AP-1 pathway, loss of RUNX2 antagonism, and proliferation and secretion of hypertrophic chondrocytes. Excessive MMP13 degrades bone trabeculae and growth plate collagen, exacerbating bone matrix loss and mineralization disorders (27). Goldner staining showed that cortical and cancellous bone mass decreased and osteoid increased, indicating that low phosphorus not only reduced new bone formation, but also weakened the normal deposition of minerals in the bone matrix. Combined with the decrease of serum alkaline phosphatase, it can be considered that there is a double abnormality of “osteogenesis deficiency combined with mineralization disorder” in the low phosphorus state, which is also an important histological basis for the increase of bone fragility, the decrease of bone mass and the change of rickets.

This study also found that the activity of osteoclasts decreased, suggesting that low phosphorus also affects the process of bone resorption (43). It is worth noting that this does not mean that low phosphorus has a protective effect on bone tissue. On the contrary, in the case of more obvious damage to osteogenesis and mineralization, even if the bone resorption activity decreased, it was still not enough to offset the bone loss and bone microstructure abnormalities caused by insufficient bone formation. Therefore, the low phosphorus group showed not a simple high-conversion or low-conversion bone metabolism, but closer to a pathological state dominated by inhibition of osteogenesis and mineralization, accompanied by imbalance of bone remodeling (44). The above results indicate that under low phosphorus conditions, bone mineralization is significantly weakened, accompanied by abnormal bone resorption.

At the level of systemic metabolism, this study observed that the levels of serum calcium, active vitamin D and parathyroid hormone in the low phosphorus group increased synchronously, suggesting that the body had obvious endocrine compensatory response under the background of continuous low phosphorus. Combined with previous understanding of the regulation of calcium and phosphorus homeostasis, this change may be related to the compensatory activation of the vitamin D-PTH axis under low phosphorus status, which aims to maintain blood calcium stability and partially improve mineral metabolism disorders (45). However, this study did not further verify the relevant endocrine regulation mechanism, and further research could be carried out in the future (46).

The mechanisms underlying impaired bone mineralization and inhibited endochondral ossification under low-phosphate conditions can be understood from multiple complementary perspectives. First, phosphate plays a dual role in skeletal mineralization: it serves both as a structural building block for hydroxyapatite crystal formation and as a signaling molecule that regulates the activity of mineralizing cells, including chondrocytes, osteoblasts, and osteoclasts-. In the low-phosphate state, this dual role is disrupted at two levels: the substrate availability for crystal nucleation and growth is directly reduced, and the cellular signaling programs that coordinate mineralization are concurrently impaired (47). Second, at the biophysical level, collagen intrafibrillar mineralization is governed by the Gibbs–Donnan equilibrium, which requires simultaneous balancing of electroneutrality and osmotic equilibrium across the collagen fibril. The reduced extracellular phosphate concentration in the low-phosphate state would directly perturb this thermodynamic equilibrium, impairing the infiltration of calcium phosphate pre-nucleation clusters into the collagen gap zones and thereby compromising intrafibrillar mineralization quality (48). This provides a biophysical explanation for the osteoid accumulation and mineralization defects observed in our Goldner staining. Third, the coordination between intrafibrillar and extrafibrillar mineralization is essential for functional bone regeneration; low-phosphate conditions disrupt not only the quantity but also the spatial organization and quality of mineral deposition (49). In addition to these biophysical and structural mechanisms, hypophosphatemia has been shown to impair hypertrophic chondrocyte apoptosis through dysregulation of PTHrP signaling and β-catenin pathways-, further contributing to the rachitic expansion of the growth plate and the failure of cartilage-to-bone transition. Collectively, these multi-level mechanisms—ranging from thermodynamic to signaling to structural—account for the impaired bone mineralization and inhibited endochondral ossification observed in our low-phosphorus model.

However, when translating the results of this study into clinical practice, it is necessary to take into account the differences between animal models and humans. This model uses extremely low phosphorus exposure (almost complete lack). Although it can effectively amplify the damage effect of low phosphorus, the phosphorus deficiency of human fetuses and premature infants is mostly insufficient intake to varying degrees, and rarely reaches a state of complete lack. In addition, there are the following important differences in calcium and phosphorus metabolism and bone development between rodents and humans (50): (1) Differences in development timing: Rats have low maturity of bone development at birth (equivalent to human fetuses in late pregnancy), while human preterm infants have more mature growth plate structure at birth, and may have different compensatory responses to phosphorus deficiency (51); (2) Placental transport mechanism: Human placenta has a high efficiency of active transport of phosphorus, and maternal mild low phosphorus may be partially buffered, while rodents have different dependence on this buffer (52); (3) the composition of breast milk: the proportion and content of calcium and phosphorus in breast milk of different species were significantly different, which affected the phosphorus intake efficiency of suckling pups; (4) endocrine regulation sensitivity: The feedback regulation threshold of human vitamin D-PTH axis may be different from rodents, and the degree of compensatory bone resorption enhancement is different (53). Therefore, the results of this study revealed the effect of maternal phosphorus deficiency on the skeletal development of offspring, but it should be combined with the characteristics of human development when extrapolated to clinical practice (54).

Our findings are of great significance for clinical work. In recent years, with the progress of perinatal medicine, the survival rate of critically ill premature infants has been increasing. As one of the common long-term complications of premature infants, the incidence of nutritional hypophosphatemic rickets has also increased. After the birth of premature infants, due to the early interruption of endogenous sources, exogenous intake is far from sufficient due to imperfect gastrointestinal function, which is prone to phosphorus deficiency. Therefore, it is of great significance to supplement phosphorus for premature infants as soon as possible to prevent the occurrence of nutritional hypophosphatemic rickets. Our study systematically revealed the damage process of nutritional low phosphorus on the long bone development of offspring rats from the aspects of biochemistry, morphology and molecular markers, that is, low phosphorus first caused the imbalance of proliferation and apoptosis of growth plate chondrocytes, then affected vascular invasion and matrix remodeling, further inhibited osteogenesis and mineralization, and finally led to delayed endochondral osteogenesis and limited bone growth. The results provide an experimental basis for understanding the mechanism of nutritional hypophosphatemic rickets in premature infants, and also suggest that clinical attention should be paid to early identification and reasonable supplement of phosphorus (32).

Of course, there are still some limitations in this study. Although the very low phosphorus model amplifies the damage effect, it is inconsistent with the actual clinical intake. There was no concentration gradient and phosphorus supplementation intervention, and the dose-effect and reversal could not be evaluated. The mechanism of osteoclast reduction and endocrine compensation is still unclear. Subsequent studies should be carried out on different low phosphorus intensity, phosphorus supplementation and pathways to be close to clinical practice (55). Finally, we fully recognize that the lack of independent gender analysis due to sample size limitations is one of the limitations of this study. In the future, it is necessary to expand the sample size to fully evaluate the potential gender-dependent programming effects.

## 5 Conclusion

In this study, the nutritional hypophosphatemia model of offspring rats was established by extremely low phosphorus diet of lactating mothers. It was found that low phosphorus status could significantly inhibit the growth and development of offspring rats and the process of endochondral ossification of long bone. Low phosphorus led to the disorder of growth plate structure, the abnormal increase of total thickness, the loss of chondrocyte polarity, the shortening of proliferation zone, the widening of hypertrophy zone, the decrease of chondrocyte proliferation and apoptosis ability, and the formation of characteristic abnormal “cartilage island” structure, which interfered with the orderly differentiation and terminal maturation of growth plate chondrocytes. In addition, low phosphorus inhibits the activity of tibial osteoblasts and osteoclasts, weakens bone mineralization, reduces bone mass and reduces cortical bone calcification. In summary, the low phosphorus state during lactation and early childhood not only directly destroys the homeostasis of growth plate tissue and the dynamic balance of chondrocytes, but also impairs bone remodeling and mineralization efficiency, eventually leading to growth retardation and abnormal bone structure. This study provides an experimental basis for understanding the mechanism of metabolic bone disease and growth disorder caused by phosphorus deficiency in premature infants (56).

## Data Availability

The original contributions presented in the study are included in the article, further inquiries can be directed to the corresponding author.
